# Real-time lane detection model based on non bottleneck skip residual connections and attention pyramids

**DOI:** 10.1371/journal.pone.0252755

**Published:** 2021-10-19

**Authors:** Lichao Chen, Xiuzhi Xu, Lihu Pan, Jianfang Cao, Xiaoming Li

**Affiliations:** 1 School of Computer Science & Technology, Taiyuan University of Science and Technology, Taiyuan, China; 2 Department of Computer Science & Technology, Xinzhou Teachers University, Xinzhou, China; Vellore Institute of Technology: VIT University, INDIA

## Abstract

The security of car driving is of interest due to the growing number of motor vehicles and frequent occurrence of road traffic accidents, and the combination of advanced driving assistance system (ADAS) and vehicle-road cooperation can prevent more than 90% of traffic accidents. Lane detection, as a vital part of ADAS, has poor real-time performance and accuracy in multiple scenarios, such as road damage, light changes, and traffic jams. Moreover, the sparse pixels of lane lines on the road pose a tremendous challenge to the task of lane line detection. In this study, we propose a model that fuses non bottleneck skip residual connections and an improved attention pyramid (IAP) to effectively obtain contextual information about real-time scenes and improve the robustness and real-time performance of current lane detection models. The proposed model modifies the efficient residual factorized pyramid scene parsing network (ERF-PSPNet) and utilizes skip residual connections in non bottleneck-1D modules. A decoder with an IAP provides high-level feature maps with pixel-level attention. We add an auxiliary segmenter and a lane predictor side-by-side after the encoder, the former for lane prediction and the latter to assist with semantic segmentation for classification purposes, as well as to solve the gradient disappearance problem. On the CULane dataset, the *F1* metric reaches 92.20% in the normal scenario, and the *F1* metric of the model is higher than the *F1* metrics of other existing models, such as ERFNet-HESA, ENet_LGAD, and DSB+LDCDI, in normal, crowded, night, dazzling light and no line scenarios; in addition, the mean *F1* of the nine scenarios reached 74.10%, the runtime (time taken to test 100 images) of the model was 5.88 ms, and the number of parameters was 2.31M, which means that the model achieves a good trade-off between real-time performance and accuracy compared to the current best results (i.e., a running time of 13.4 ms and 0.98M parameters).

## Introduction

The rapid growth of car ownership has caused an escalating conflict between vehicles and road resources, and the complexity of road conditions and the not yet fully mature communication and intelligent driving technologies make the safety of car driving an increasingly important issue. To this end, Alazab et al. used an improved Dijkstra algorithm to accomplish optimal transport path selection for dynamic traffic flow [1]. Javed A R et al. used a CANintelliIDS model that fuses convolutional neural networks and attention-gated recurrent units (GRUs) to detect single and mixed intrusion attacks on the CAN bus to ensure the security of in-vehicle communication [2]. ADAS can guarantee the safety of vehicle driving with the aid of vehicle sensors to perceive external conditions. Lane detection, an indispensable component of ADAS, plays an essential role in departure warning, lane keeping, and trajectory planning. Lane detection in complicated traffic scenes is often perceived as a highly challenging task. First, sensor-generated data from the vehicle are subject to anomalies caused by faults, errors, and/or cyberattacks and need to be detected accurately. Second, the lane line characteristic information is heavily weakened in scenarios such as scenarios involving light changes, road damage, and object occlusion, which makes the accuracy of lane line detection poor. Finally, ADAS have high demands for real-time lane line detection, making it difficult to simultaneously fulfill the real-time and accuracy requirements.

The combination of a multistage attention mechanism and a convolutional neural network (CNN) with long short-term memory (LSTM) has efficiently reduced the number of anomalous instances in the dataset, and this has removed obstacles to data collection for related tasks such as lane line detection [3]. At present, there are two types of vision-based lane detection methods: traditional methods and deep learning methods. The available traditional lane detection algorithms based on hand-designed features extract the color, edge, texture and shape of lanes through a color histogram, Sobel algorithm, LBP algorithm, SIFT algorithm or Hough transform and combined lane marker grouping [4]; then, they output lane lines from straight lines or curve model fitting via a mathematical model. Although the calculations performed by traditional methods are extremely simple, there are still shortcomings in many complex road scenarios, such as a lack of lines, blocked lanes, and poor light. Therefore, traditional methods are no longer able to meet the substantial requirements of autonomous vehicle driving [5]. The development of deep learning has opened new horizons for lane line detection. It extracts rich information and has a superior model robustness, which compensates for the shortcomings of traditional algorithms to some extent, but the real-time performance and accuracy of detection cannot satisfy the requirements of intelligent driving in complex scenarios such as scenarios involving object occlusion and shadow interference environments.

According to the strengths of semantic segmentation algorithms in traffic scenario parsing, we improve the ERF-PSPNet semantic segmentation model, which uses the non bottleneck skip residual connections (Non-bt-1D-SRC) module in the encoder stage to integrate abundant convolutional layer information, and the decoder uses the IAP module to minimize the number of parameters and extract rich contextual information. Under multiscene environment interference, the real-time performance and accuracy of lane line detection have been improved, and the limitations of available algorithms have been effectively overcome.

## Related work

Traditional lane detection algorithms based on hand-designed features are generally divided into four steps: (1) lane marking generation, (2) lane marking grouping, (3) lane model fitting, and (4) temporal tracking [6]. The lane image is captured by a camera located behind the windshield, and lane line detection uses lane markers to locate whether the vehicle is well inside the lane boundary. Li et al. [7] proposed a lane detection algorithm based on a line segment detector (LSD) and a weighted hyperbolic model to determine the effect of inverse perspective mapping (IPM) on lane detection, to reduce the noise generated by lane markings and shadows, and to divide lane detection into near-field line detection and far-field curve fitting. Lee et al. [8] proposed an efficient and robust lane detection and tracking algorithm that uses the region of interest (ROI) of an input image to reduce redundant image data; the algorithm is divided into three steps: initialization, lane detection, and lane tracking. Hu et al. [9] proposed a new method of lane detection combined with model predictive control for effective lane information extraction and trajectory tracking by using a dynamic ROI extraction method based on longitudinal vehicle speed changes to improve the real-time performances and adaptability of traditional image information extraction methods. In a recent study, lane lines were detected with perspective transformation, threshold processing, mask operations and sliding window optimization [10]. These algorithms rely on intuitive means and mathematical knowledge and are only applicable to a single scene environment; however, it is not easy to obtain continuous edge features in real-time traffic scenes with uneven illumination and obstacle occlusion when lane lines have broken edges and discontinuous brightness.

With the advent of convolutional neural networks and the rapid development of the computing power of hardware, deep learning has demonstrated its evident nature and competitiveness in solving many computer vision problems. Gadekallu TR et al. utilized crow search algorithms for hyperparameter tuning of CNNs and achieved excellent performance in gesture recognition [11]. Vasan et al. implemented image-based malware classification with the help of a CNN [12]. Scholars at home and abroad have also applied CNNs to detect lane lines [13, 14] to address the challenges that traditional detection algorithms encounter in multiple scenarios. Liu et al. [15] conceived a label-guided attentional distillation (LGAD) method for lane line segmentation that separately considered lane labels and target images as inputs to the teacher network and student network and employed the teacher network to reinforce the attentional map of the student network. However, substantial computational resources are required to train the teacher network. Liu et al. [16] presented style transformation for data augmentation to generate images in low-light conditions with generative adversarial networks that improve the environmental adaptability of the lane detector, which does not demand any additional manual annotation or inference overhead. Yun et al. [17] used the horizontal reduction module to compactly extract the lane marker information in the image and achieved end-to-end lane marker detection via row-wise classification. Liu et al. [18] proposed a multitask fusion lane line detection model that utilizes semantic segmentation to extract lane features and heat map regression to predict the vanishing point of lanes. Lee et al. [19] introduced an extended self attention (ESA) module, which is divided into horizontal ESA (HESA) and vertical ESA (VESA). Each module extracted the occlusion location by predicting the confidence of the lane in the vertical and horizontal directions, and the model is robust in occluded and low-light environments. Li et al. [20] applied a modified encoder-decoder network with an instance-batch normalization net (IBN-NET) and an attention mechanism based on the LaneNet structure, which is well suited for two types of semantic segmentation (SS) tasks with only lanes and backgrounds, but further improvement is needed regarding the extraction of road environment structures; to this end, Ye et al. [21] proposed a new method of describing roads using waveforms to analyze the local and global features of road geometries to detect lane markings. To some extent, these algorithms compensate for the shortcomings of traditional algorithms regarding lane detection in complex scenarios, but their real-time performances and accuracies in terms of detection remain poor. To this end, we propose a model that fuses Non-bt-1D-SRC and attention pyramid (AP) for real-time lane detection; this model not only extracts abundant contextual scenarios but also satisfies the demands of real-time performance and accuracy in intelligent driving with fewer parameters and better detection outcomes than existing methods.

Related work presented in the literature and the main methods, innovations and limitations are shown in Table 1.

**Table 1:** Summary of the related work.

	Paper cited	Main methods	Innovations	Limitations
	Li et al. [7]	A hyperbolic model	Near field: LSD + DBSCAN.	Failure to fit the S-lane well.
			Far field: hyperbolic model.	
	Lee et al. [8]	A lane detection algorithm with an efficient ROI	VP with clustering and tracking scheme, EDLines, and Kalman filter in ^-ROI.	Cannot detect lanes on snowy and rainy nights.
Traditional algorithms				
	Hu et al. [9]	A novel approach that combines lane detection and model predictive control	Dynamic ROI extraction, edge and Hough detection.	Needs a good mathematical foundation.
	Liu et al. [15]	ENet_LGAD	Uses a teacher network to reinforce the attentional map of the student network.	Requires high computational resources.
	Liu et al. [16]	SIM_CycleGAN +ERFNet	Style transfer and generative adversarial networks.	Performs poorly except in low light.
	Yun et al. [17]	ERFNet_E2E	Uses horizontal reduction to extract information.	Fails to detect lanes when reflections exist.
Deep learning algorithms				
	Liu et al. [18]	ERFNet-VP	SS and heat map regression.	Poor lane line fitting.
	Lee et al. [19]	ERFNet-HESA	An ESA, which is divided into an HESA and a VESA.	Less effective for datasets with fewer occlusions.
	Li et al. [20]	Improved SS	IBN-Net and attention mechanism.	Sensitive to the structure of the road environment.
	Ye et al. [21]	Based on lane structural analysis and CNNs	Waveforms to analyze the local and global features to detect lane markings.	Additional parameters are needed to control deviations.

Our main contributions can be summarized as follows:

- We design a skip residual connection module that joins the given input features with the residual features of multiple layers in a non bottleneck module to solve the problem regarding the lack of relevant characteristic information for adjacent convolutional layers in non bottleneck modules.
- We propose an improved decoder structure by adding an AP, which prominently decreases the number of parameters utilized and enables us to extract abundant global contextual information from images.

## Methods

**Fig 1:**
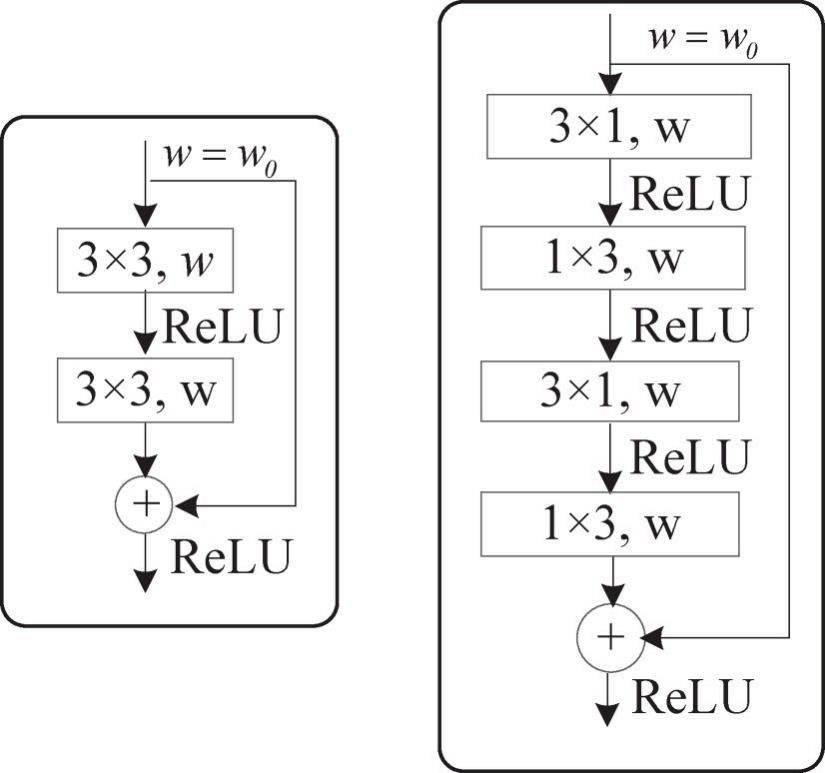
Diagram of the bottleneck residual block and Non-bt-1D module, where *w*_*0*_ denotes the number of channels in the upper layer output and *w* denotes the number of channels in the input.

The bottleneck module, a basic structure proposed by He et al. [22], increases the depth and decreases the computational complexity of a network, but it is always subject to degradation problems [23]. Romera et al. [24] introduced non bottleneck-1D (Non-bt-1D) modules, which use 1D factorization to accelerate and reduce the number of parameters in the original non bottleneck layer. It disaggregates the 3×3 convolution of a bottleneck residual module into combinations of 3×1 and 1×3 convolutions, reducing the number of network parameters by 33%; this is equal to the decomposition of two 3×3 convolution kernels in the regular residual module into two sets of 3×1 and 1×3 one-dimensional convolutions [25]. The fusion of the input features and the convoluted feature maps using residual connections also strengthens the expressiveness of the network. These modules are shown in Fig 1.

### Non-bt-1D-SRC module

The output of the Non-bt-1D module of the ERFNet encoder is determined by only the input features and output features; however, there are multiple convolutions inside the Non-bt-1D module, and if only the input and output features are connected, the intermediate features are likely to be lost. Although ERF-PSPNet [26] fuses the encoder of ERFNet and the decoder of PSPNet [27], the decoder tends to be more complicated, and less contextual information can be extracted. In view of this, a lane detection model that fuses Non-bt-1D-SRC with an AP is presented below as a reference.

**Fig 2:**
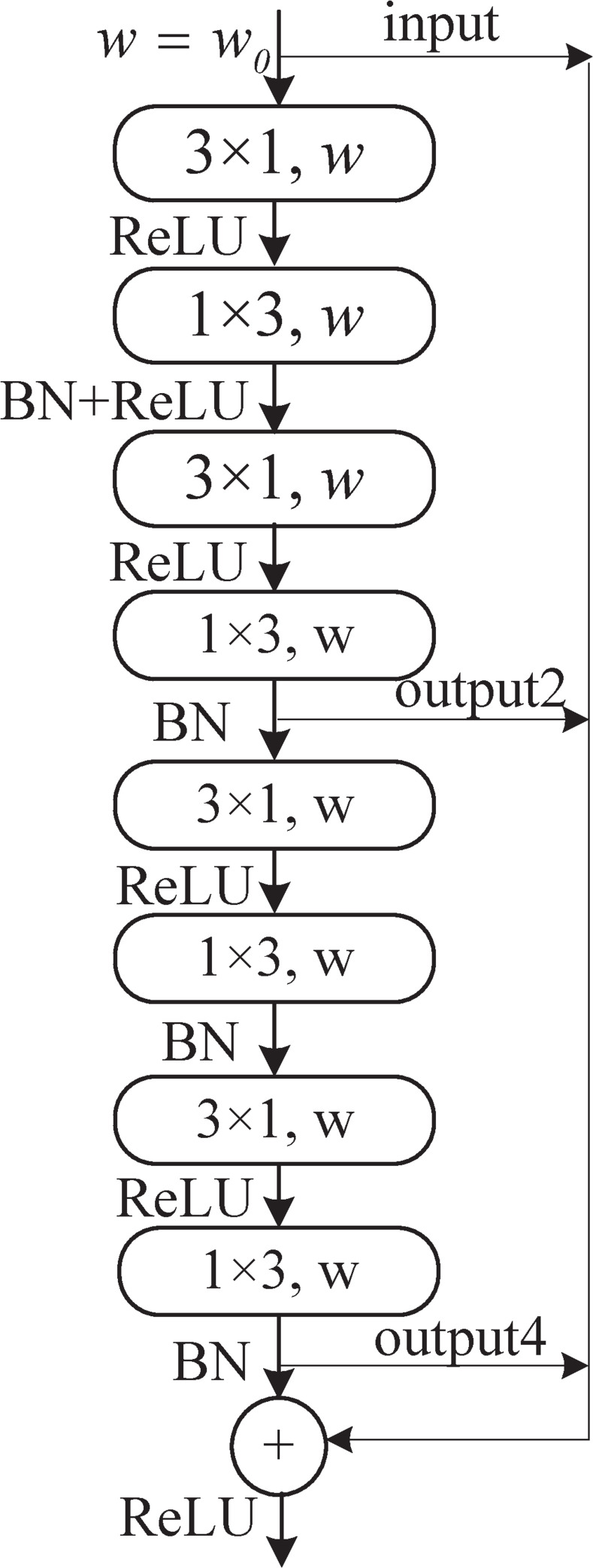
Non-bt-1D-SRC module, where *w*_*0*_ is the number of output channels in the last layer; here, *w*_*0*_ is 128. Input, output2, and output4 denote the input features together with the output features after two pairs of 3×1 and 1×3 combination operations.

Zhao et al. [28] studied a multilevel skip residual connection block to overcome the problem of a lack of relevance between adjacent convolutional layers, and their approach achieved excellent results on image superresolution reconstruction tasks. Accordingly, we design a Non-bt-1D-SRC module to resolve the problem regarding the lack of relevant characteristic information between neighboring convolutional layers in the non bottleneck module. Our module cross-stacks 2 sets of 3×1 and 1×3 convolution blocks (the first 3×1 convolution operation is followed by ReLU) and adds batch normalization (BN) to accelerate the training of the neural network and reduce the dependence of the gradient on the model parameters after the 1×3 convolution [29]. The 2nd to 4th 3×1 and 1×3 convolution blocks adopt dilation rates of 2, 4, and 8, respectively, for dilated convolution to collect additional background information while employing random deactivation to prevent overfitting. The features after each pair of 3×1 and 1×3 convolution blocks are subblocks of the residual connection and are summed with the convolution result to obtain the final result. The non bottleneck skip residual connection module is shown in Fig 2.

### IAP module

An attention mechanism [30] enables humans to allocate limited computational resources to focus on regions of interest when processing complex visual information, providing more easily processed and relevant information for more complex visual processing tasks [31]. Incorporating an attention mechanism in a neural network is an efficient technique to tackle resource allocation in a problem with information overload.

**Fig 3:**
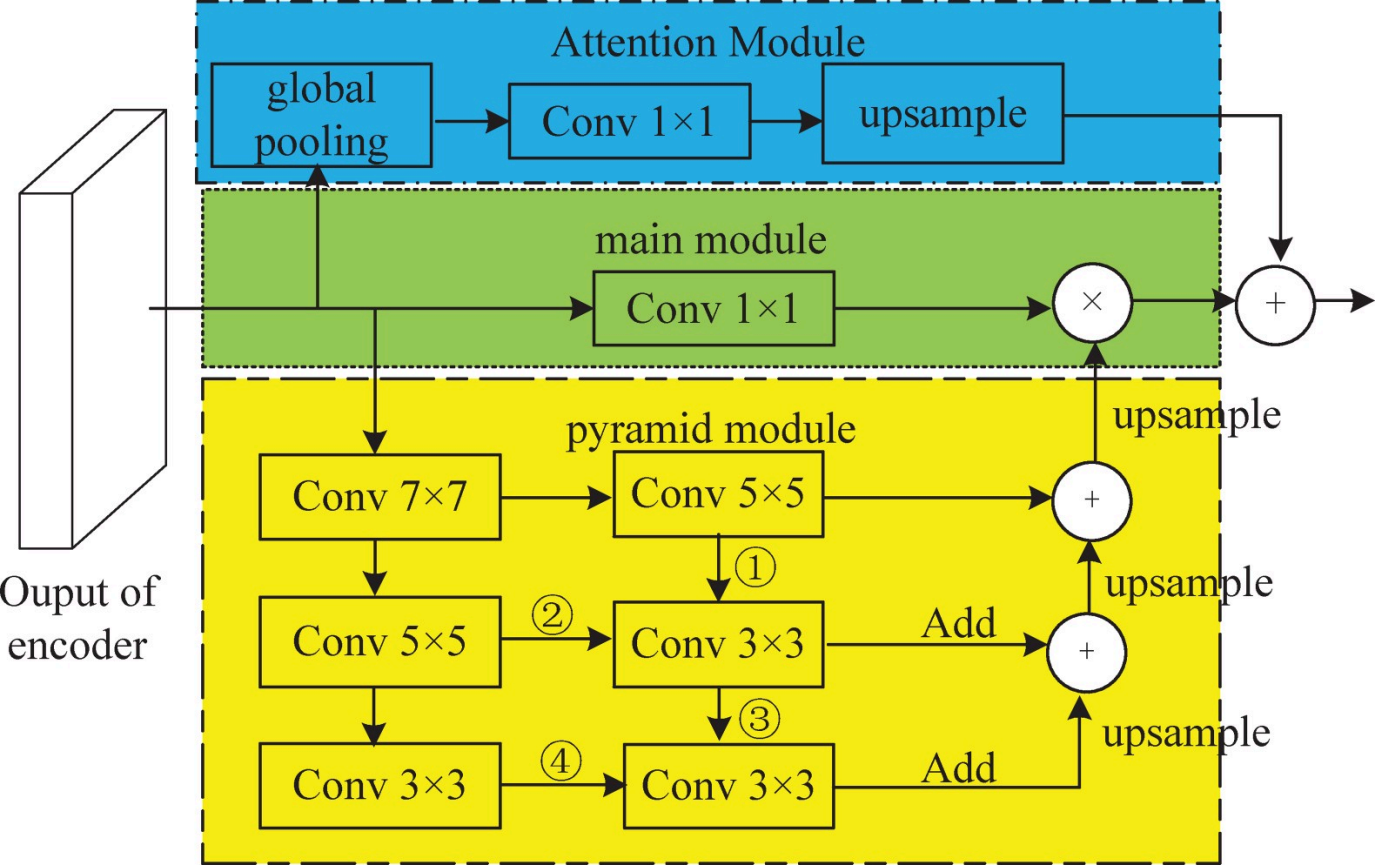
AP and IAP modules.

The decoder of ERF-PSPNet utilizes a pyramid pooling module (PPM) to effectively converge the information obtained from different subregions [32]. The contextual information extracted from this approach is very limited, whereas the AP model can exploit abundant features to extract and evaluate the semantic labels on each pixel. However, the available AP modules contain more parameters and extract less contextual information. For this reason, we employ an IAP module, which includes three parts, namely, a main module, a pyramid module, and an attention module, and the AP and IAP models are shown in Fig 3A and 3B.

The main module performs a 1×1 convolution on the encoder output; the attention module adopts global pooling, executing 1×1 convolution and upsampling operations on the encoder output features; we remove the second 7×7, 5×5, and 3×3 convolutions from the original AP module and replace them with 5×5, 3×3, and 3×3 convolutions while joining them in pairs to form a small pyramid network. The results of ③ and ④ are added after a 3×3 convolution and then upsampled; ① and ② use the same operation as ③ and ④. In the IAP module, there are two pyramid networks that considerably reduce the number of channels and parameters and decrease the complexity of the overall network. The output of the main module is multiplied piecewise with the output of the pyramid module, and the result is then added to the output of the attention module.

### Improved lane detection model

**Fig 4:**
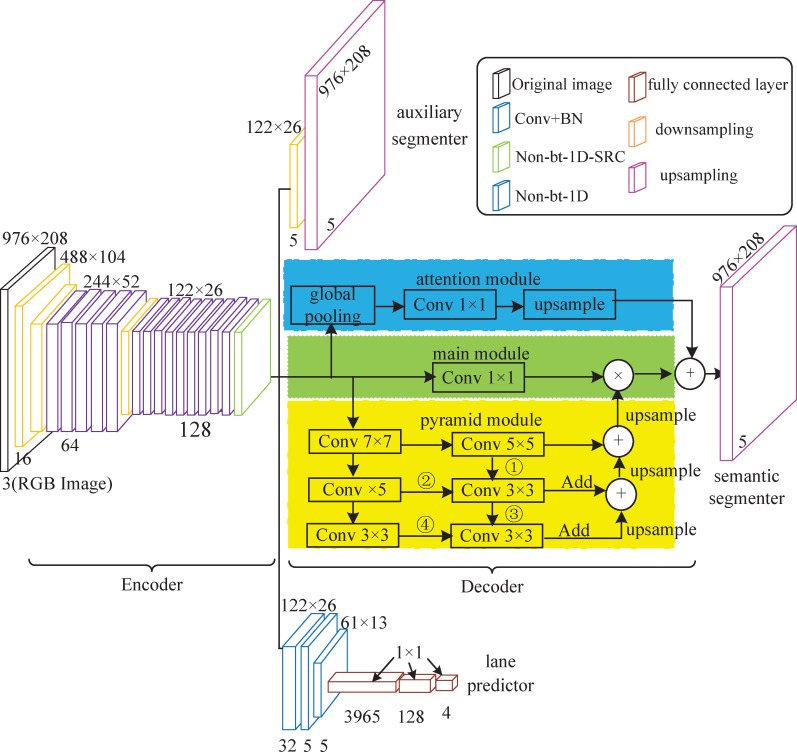
The Nb_SINet lane detection model.

The structure of the improved lane detection model is shown in Fig 4.

The model consists of three components: an auxiliary segmenter (Its loss is shown in S1 Fig), a lane predictor [33] (for predicting the presence of lanes, its loss is shown in S2 Fig), and a semantic segmenter (for solving the vanishing gradient problem, its loss is shown in S3 Fig). The semantic segmenter, based on an encoder-decoder prototype, extracts enriched landscape features through downsampling and Non-bt-1D and Non-bt-1D-SRC operations. The decoder introduces the IAP module and adds the designed attention mechanism, where the decoder, auxiliary splitter and lane predictor operate side by side. The model is named the "Non-bt-1D-SRC_IAP network" and is abbreviated as "Nb_SINet".

### Lane detection process

**Fig 5:**
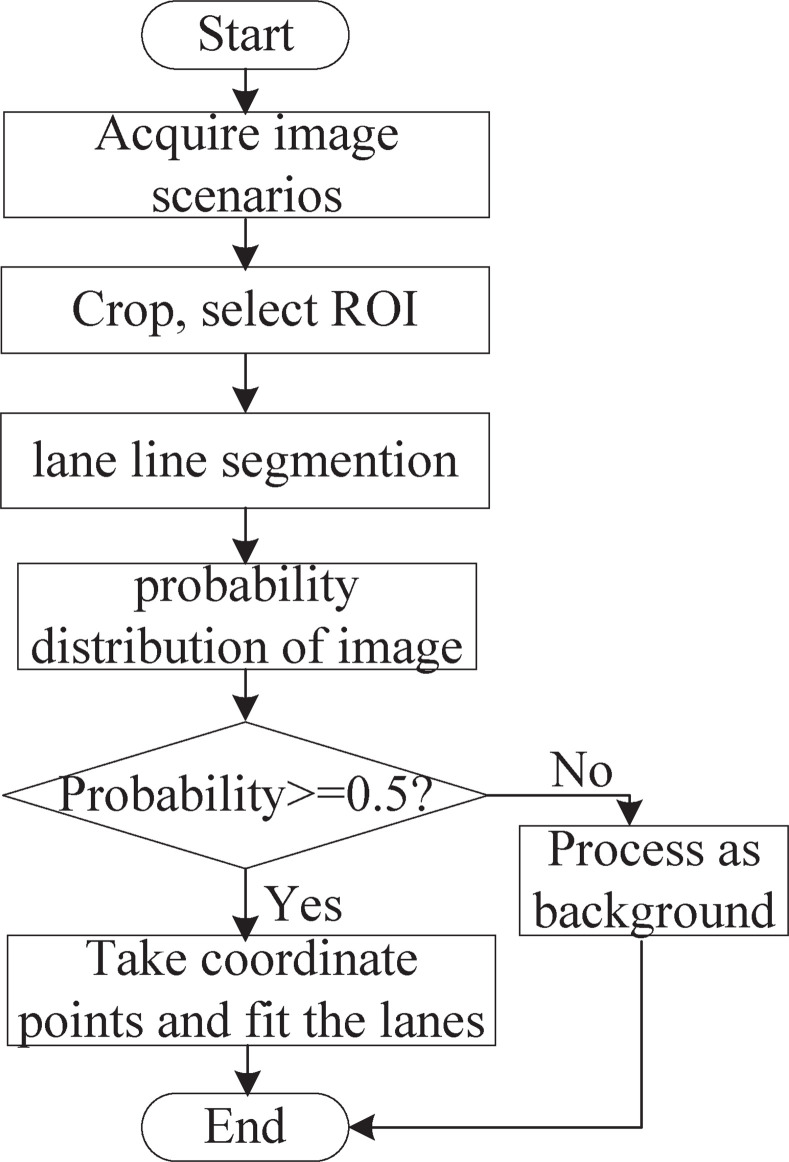
Flow chart for lane detection. The process involves three steps, including image preprocessing, lane line segmentation and lane line fitting.

The proposed lane detection process is shown in Fig 5.

We obtain the ROI of the input image using OpenCV and feed the image into the Nb_SINet model for SS after a series of preprocessing steps, including cropping and rotation, to obtain the lane line probability distribution map. If the probability is greater than or equal to 0.5, point fitting is carried out on the lane line; otherwise, the line is processed as background.

## Experiments

### Dataset

**Fig 6:**
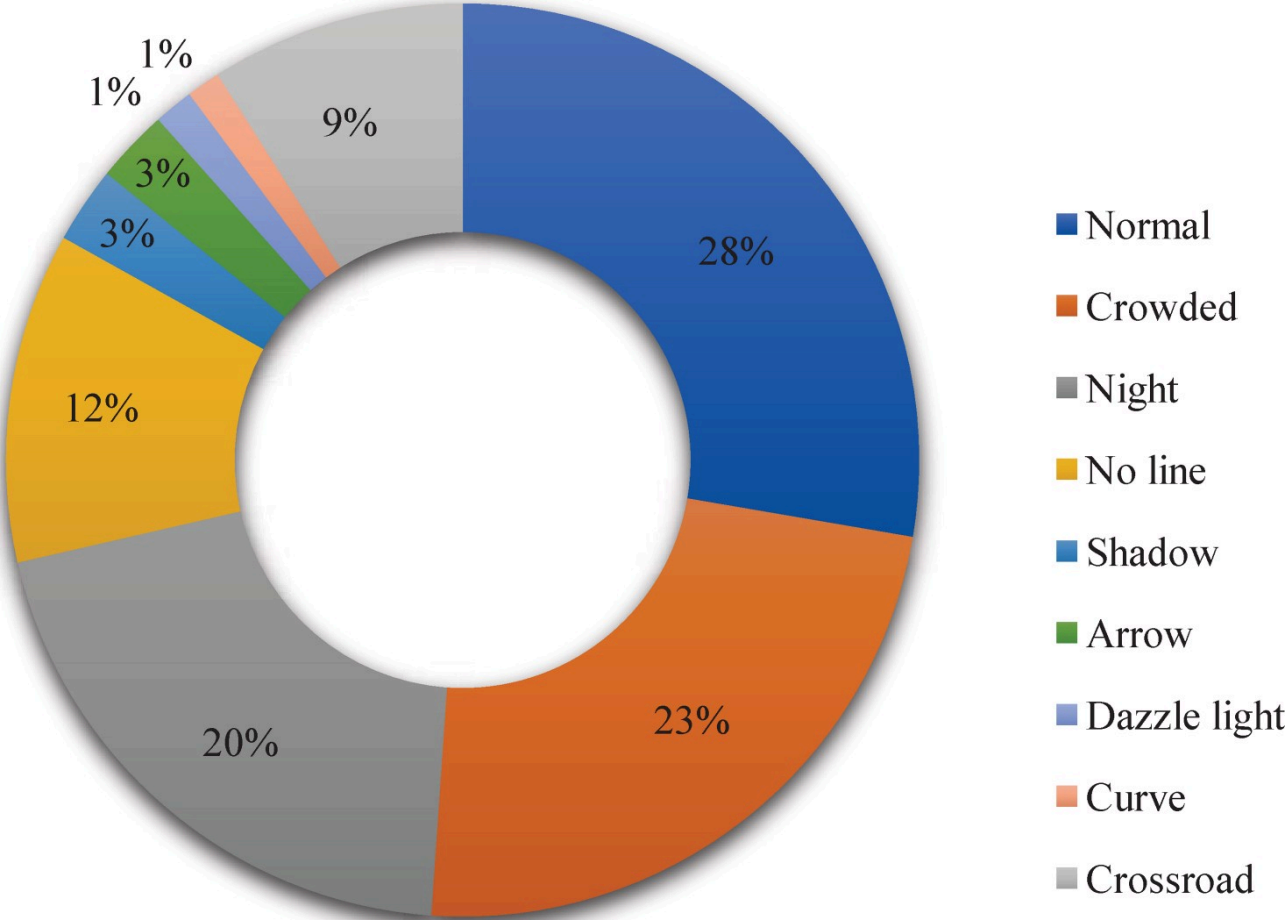
Scenarios and their proportions.

To verify the robustness of our model in complicated scenarios, we use the public dataset CULane [34] in our experiments. It contains 133,235 images at resolutions of 1640 × 590, including 88,880 training images, 9675 validation images, and 34,680 testing images. The images were captured from nine different scenarios by cameras mounted behind the front windshields of six vehicles, and the proportion of each category is shown in Fig 6.

We select the ROI as the region in the image containing lane lines, crop the image to a resolution of 976×208, and apply random scaling and random rotation to enlarge the dataset while strengthening the robustness of the model.

### Evaluation metrics

Each lane line is marked in the dataset as a line with a width of 30 pixels according to the literature [35]. Then, we calculate the intersection over union (IoU) between the real lane line and the predicted lane line, where an IoU greater than 0.5 is regarded as a true positive. The mean IoU (mIoU) refers to the mean of IoUs for all categories, and the equation is as follows [36]:

$$mIOU=\frac{1}{k+1}\sum_{i=0}^{k}\frac{TP}{FN+FP+TP}$$ (1)

We assume that there are k+1 classes (including an empty class and a background class); *TP*, *FP* and *FN* are the numbers of true positives, false positives and false negatives, respectively [37]. Precision indicates how many of the samples with positive predictions are actually positive samples. Recall indicates the probability that a positive sample is correctly predicted in the original positive sample. *F1* represents the harmonic mean of precision and recall. The *FP* metric is measured for the crossroad scenario, and the *F1* metric is measured for the rest of the scenarios. Precision, recall and *F1* are calculated as follows [38]:

$$Precision=\frac{TP}{TP+FP}$$ (2)

$$Recall=\frac{TP}{TP+FN}$$ (3)

$$F1=\frac{2\cdot Precision\cdot Recall}{Precision+Recall}$$ (4)

### Implementation details

In this experiment, we train our model on a machine equipped with an Ubuntu 16.04 LTS 64-bit operating system and two GeForce GTX 1080 GPUs containing 12 GB of memory; the model is implemented in Python (the Pytorch framework), and then, we calculate the final results with MATLAB. We use TensorboardX to visualize the induced loss variation.

We use the optimal parameters of ERFNet on the Cityscapes dataset as pretraining parameters for transfer learning, and we apply the SGD optimizer with an initial learning rate of 0.01 and a batch size of 4. The number of iterations is 6.66×10^5^. We adjust the learning rate based on the loss value until the loss does not change for several consecutive epochs.

**Fig 7:**
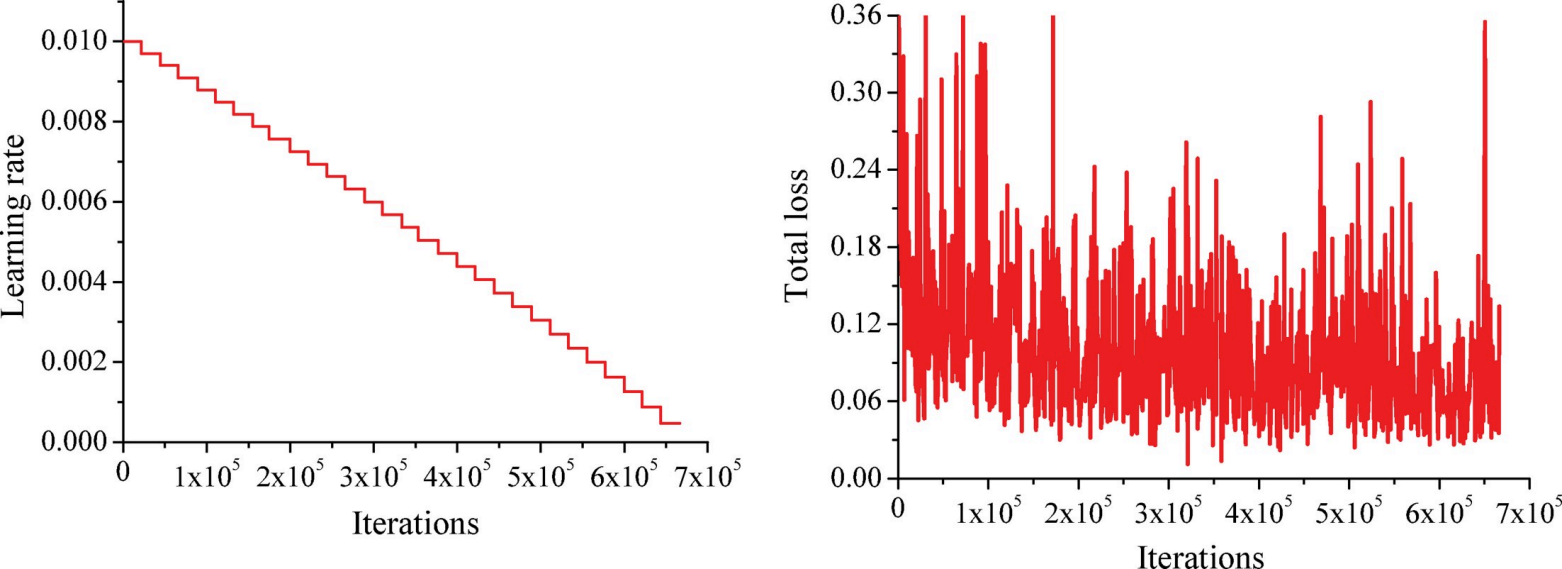
Graphs of the learning rate and total loss. (a) Learning rate. (b) Total loss.

The learning rate and total loss (the weighted sum of the SS loss, auxiliary segmentation loss, and lane prediction loss) curves yielded during the training process are shown in Fig 7.

The total loss reaches 0.073 when the number of iterations reaches 6.548 ×10^5^. The total loss converges faster than the learning rate.

**Fig 8:**
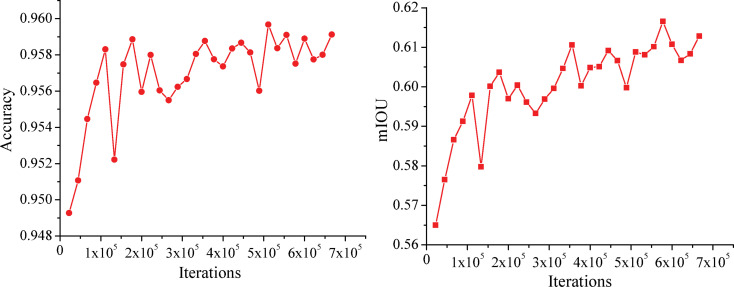
Graphs of the accuracy and mIoU achieved on the validation set. (a) Accuracy. (b) mIoU.

The accuracy and mIoU curves obtained on the validation set are shown in Fig 8A and 8B, respectively. The accuracy reaches 95.94%, and the mIoU reaches a maximum value of 61.55% when iterating 5.555×10^5^ times.

### Comparison and analysis

In this section, we prove the effectiveness of the Non-bt-1D-SRC module and the IAP module by conducting ablation experiments, and we prove the superiority of our model by a comparison with existing models.

#### Effectiveness of the Non-bt-1D-SRC module

In this section, we perform experiments to verify the significant effect of the skip residual connection module. The *F1* metrics (and *FP* metrics at crossroads) obtained for each scenario with the encoder using Non-bt-1D versus those obtained using our Non-bt-1D-SRC module are shown in Table 2, where both decoders use a PPM.

**Table 2:** Comparison between the results obtained using the Non-bt-1D and Non-bt-1D-SRC encoders.

Scenarios	Non-bt-1D	Non-bt-1D-SRC
Normal	91.84	**92.11**
Crowded	**72.01**	71.47
Night	69.13	**69.82**
No line	46.34	**46.84**
Shadow	**67.06**	63.19
Arrow	**87.39**	87.23
Dazzle light	64.26	**65.96**
Curve	**69.21**	67.75
Crossroad (FP)	2,590	**2,469**
Mean *F1*	73.70	**73.76**

From Table 2, the performances of the Non-bt-1D-SRC module are better than those of the Non-bt-1D module in the normal, night, no line, dazzle light, and crossroad scenarios, and the mean *F1* improves for the nine scenarios, thereby proving the effectiveness of the Non-bt-1D-SRC module through ablation experiments.

#### Effectiveness of the IAP module

**Fig 9:**
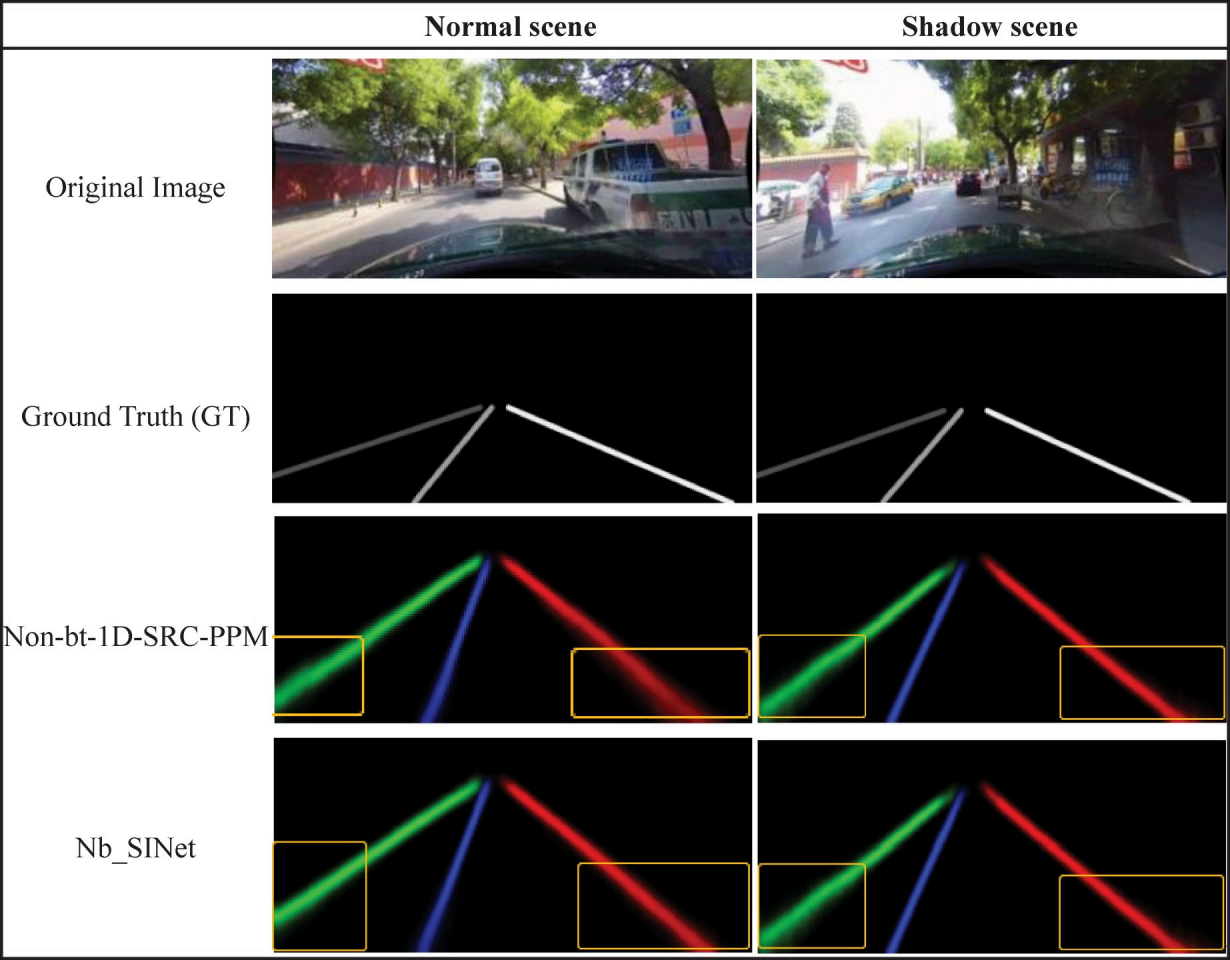
Semantic segmentation diagram of lane lines.

Based on the above experiments, we randomly choose several scenarios to compare the lane line detection probability distribution plots obtained when the AP (named the Nb_SAP model) and IAP (named the Nb_SINet model) modules are used as decoders, and the semantic segmentation diagram of lane lines before and after improvement (introducing both auxiliary trainers and lane predictors) are shown in Fig 9.

Fig 9 lists the original images in the normal and shadow conditions in each scene, as well as the ground truth, Non-bt-1D-SRC-PPM and the semantic segmentation diagram of the Nb_SINet model.

In Fig 9, two kinds of images under normal and shadow conditions are randomly selected, and the fitting plots of the original image obtained when the AP module and the IAP module from this paper are used as the decoder are listed in turn, along with the real labeling of each image. After using the AP as the decoder, the probability distribution of the lane lines in the yellow box is more discrete; however, after using the IAP, the probability distribution of the lane lines is more concentrated, and the fitted lane lines are closer to the real labeled image, which indicates the effectiveness of the IAP.

Table 3 shows the changes in the *F1* and *FP* (crossroad only) test metrics of the CULane dataset before and after improving the decoder. Nb_SAP is the model using Non-bt-1D-SRC and the AP as the encoder-decoder.

**Table 3:** Comparison between the *F1* and *FP* metrics of Nb_SAP and Nb_SINet.

Scenarios	Nb_SAP	Nb_SINet
Normal	92.11	92.24
Crowded	72.10	73.36
Night	69.22	69.08
No line	47.20	47.17
Shadow	67.55	74.82
Arrow	87.67	87.61
Dazzle light	65.09	68.18
Curve	68.40	68.24
Crossroad (FP)	2,881	2,664
Mean F1	73.76	74.10

The F1 index improves in the normal, crowded, shadow, dazzle light, and crossroad scenarios after the decoder adopts the IAP model, especially in the shadow scenarios, where the index improves remarkably. The mean *F1* score for the nine scenarios increases noticeably.

**Fig 10:**
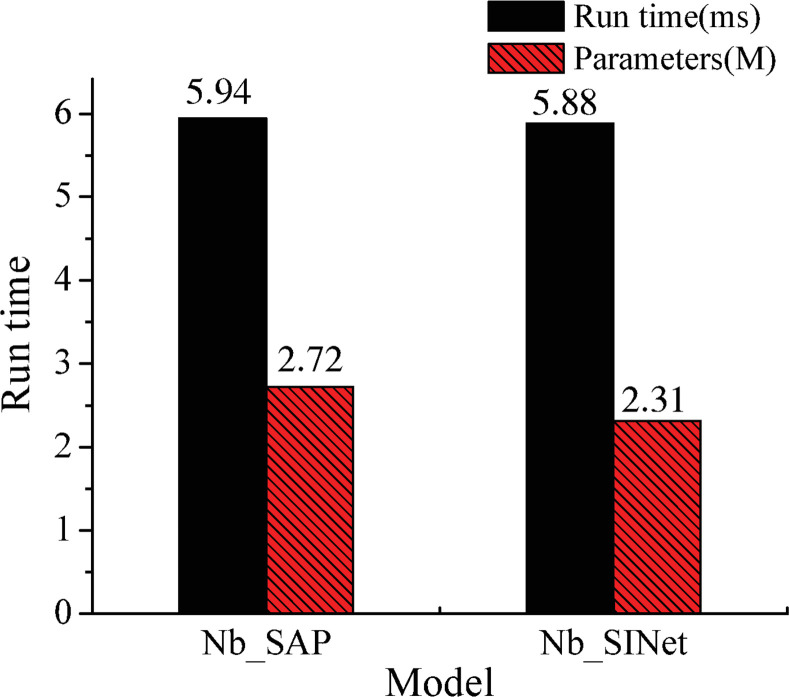
Comparison between the runtime and parameters of the two tested models.

The number of parameters and runtime are shown in Fig 10 before and after improvement.

The number of parameters is reduced by 0.41M and the runtime is reduced by 0.06 ms when the IAP module is used as the decoder; this verifies the effectiveness of the IAP module in terms of reducing the number of parameters and the runtime.

#### Ablation experiments with assisted trainers

In this section, we conduct ablation experiments on the model with the auxiliary trainer (named Nb_SINet) and without the auxiliary trainer (named Nb_SINet_noaux), and the metric comparison for each scenario is shown in Table 4.

**Table 4:** Performance comparison of auxiliary trainer ablation experiments.

Scenarios	Nb_SINet_noaux	Nb_SINet
Normal	92.07	92.24
Crowded	72.10	73.36
Night	68.90	69.08
No line	47.45	47.17
Shadow	70.05	74.82
Arrow	88.22	87.61
Dazzle light	65.13	68.18
Curve	68.58	68.24
Crossroad (FP)	2,797	2,664
Mean F1	73.86	74.10
Parameters (M)	2.31	2.31
Runtime (ms)	5.88	5.88

In Table 4, the *F1* indexes in the normal, crowded, night, shadow, dazzle light, and curved scenes obviously increase after incorporating the auxiliary trainer, the number of *FPs* in the crossroad scenario is clearly reduced, and the mean *F1* increases from 73.86 to 74.10. Additionally, the effects of incorporating the auxiliary trainer on the runtime and the number of parameters are small, which indicates that incorporating the auxiliary trainer helps with the training process of the model, thereby proving the usefulness of the auxiliary trainer.

#### Comparison with available models

To verify the effects of our model, we undertook a broad comparison with several state-of-the-art methods. We evaluated Nb_SINet and multiple backbones, i.e., ENet_LGAD [15], SIM_CycleGAN+ERFNet [16], ERFNet-E2E [17], ERFNet_VP [18] and ERFNet-HESA [19], for each scenario, and the mean *F1* for each method is also shown in Table 5.

**Table 5:** *F1* index for each model in different scenarios.

Scenarios	ENet_LGAD	SIM_CycleGAN+ERFNet	ERFNet-E2E	ERFNet-HESA	ERFNet_VP	Nb_SINet
Normal	91.2	91.8	91.00	92.0	91.9	**92.24**
Crowded	68.9	71.8	73.10	73.1	72.3	**73.36**
Night	66.8	69.4	**73.10**	69.2	69.4	70.20
Shadow	66.4	**76.2**	74.10	75.0	74.0	74.82
Arrow	84.5	87.8	85.80	**88.2**	87.4	87.61
Dazzle light	59.9	66.4	64.50	63.8	67.1	**68.18**
Curve	66.4	67.1	**71.90**	67.9	66.4	68.24
No line	43.7	46.1	46.60	45.0	46.8	**47.17**
Mean F1	72.0	73.9	74.00	**74.2**	**74.2**	74.10

Nb_SINet achieves excellent performance, with an F-measure of 74.10%, and outperforms other methods in almost all categories. There are noticeable performance improvements in the dazzling light and no line scenes.

**Fig 11:**
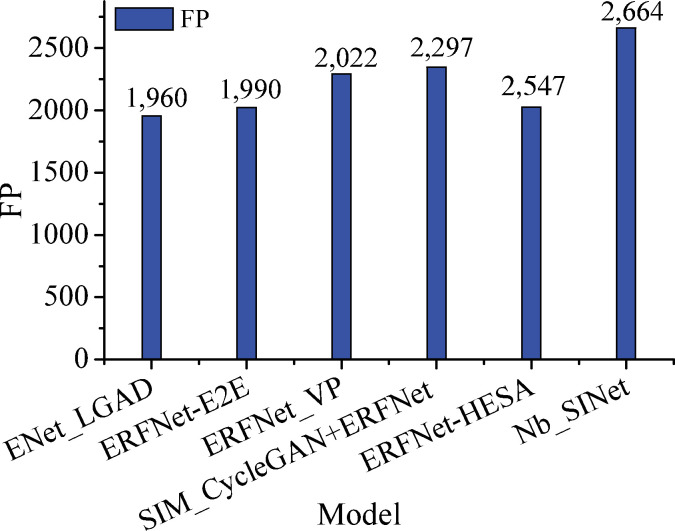
Comparison of the *FP* results obtained for the crossroad scenario.

The comparison results regarding the *FP* metrics for each model in the crossroad scenario are shown in Fig 11.

The *FP* of our model in the crossroad scenario is 2,664, while that of the ENet_LGAD model is only 1,955, so the latter model requires further improvement for the crossroad scenario.

**Fig 12:**
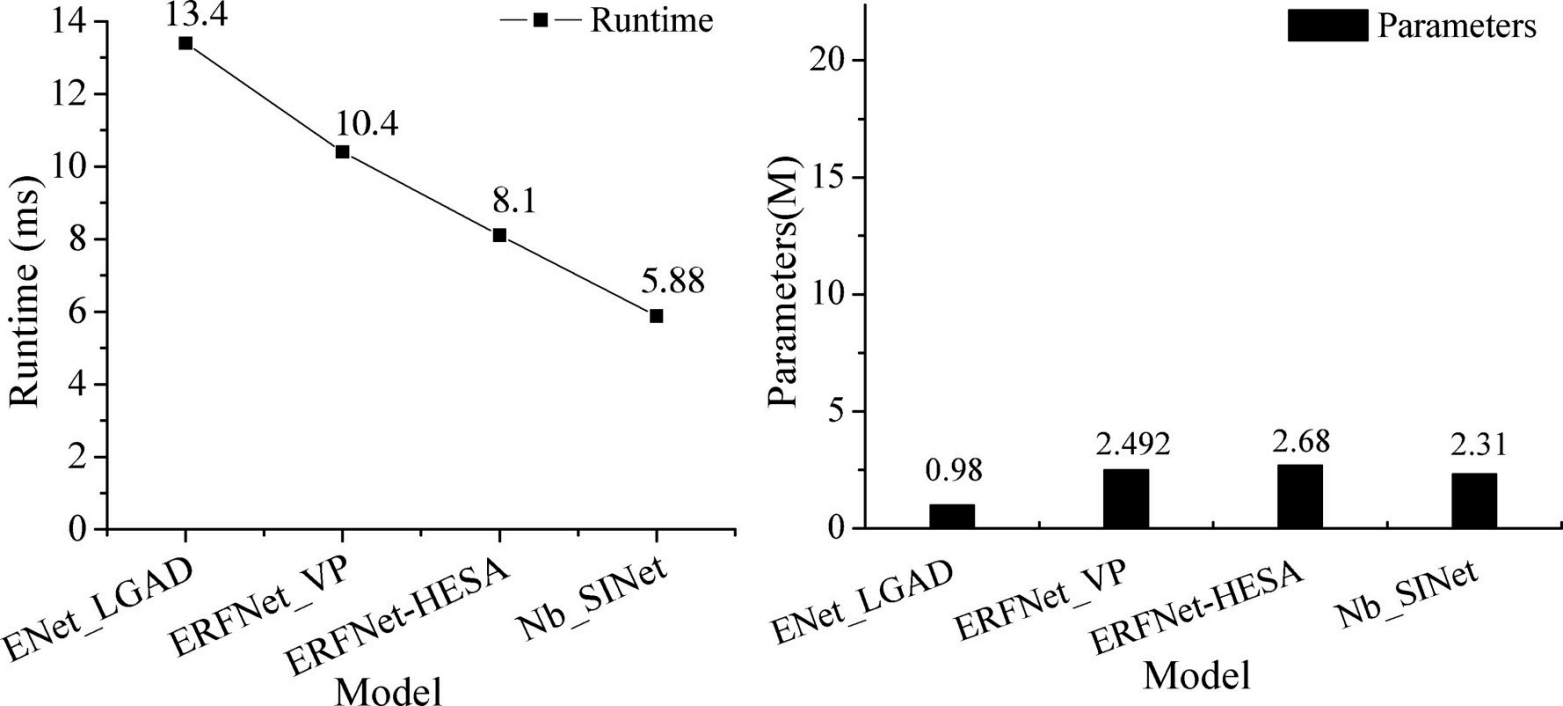
Comparison between the runtime and numbers of parameters for the tested models. (a) Comparison of runtimes. (b) Comparison of the number of parameters.

We compare our model with the ENet_LGAD, ERFNet_VP, and ERFNet-HESA models in terms of both their runtime and numbers of parameters, and the comparison results are shown in Fig 12.

In Fig 12, the runtime of our model is only 5.88 ms, and the number of parameters is only 2.31M, so our model outperforms the state-of-the-art ENet_LGAD in terms of accuracy and number of parameters. Our model meets the state-of-the-art requirements, satisfying the trade-off between the number of parameters and runtime.

### Lane line fitting outcomes

**Fig 13:**
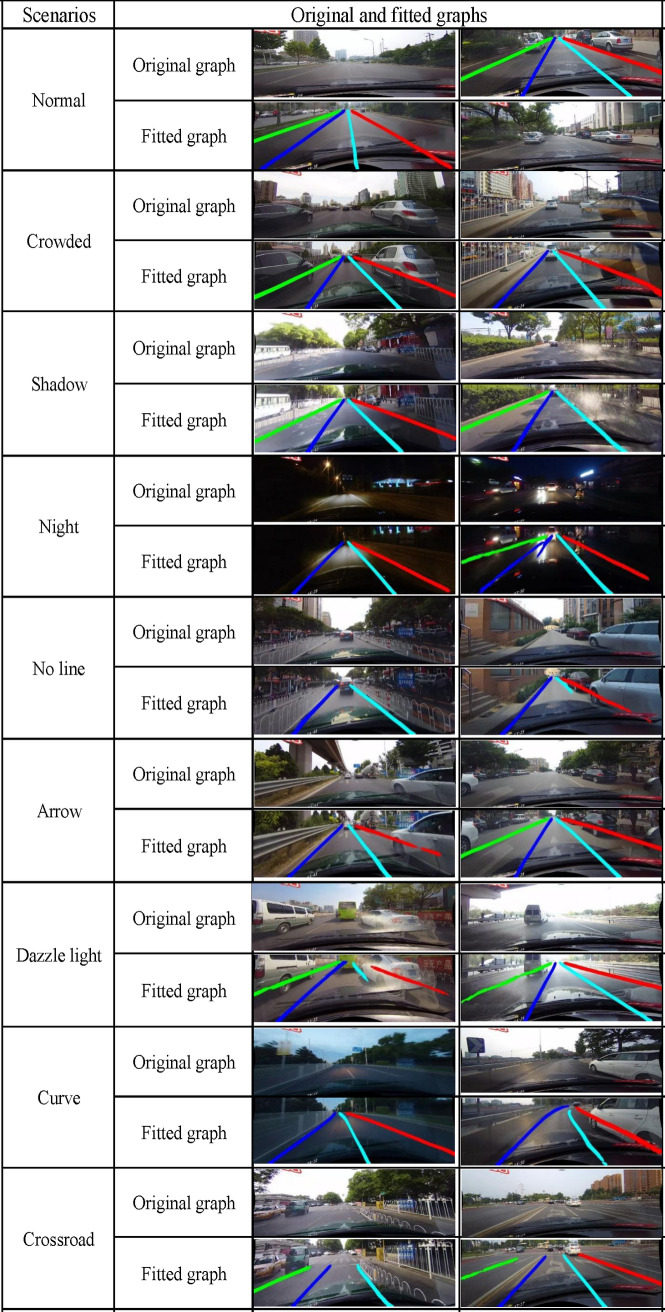
Original drawings and diagrams with the fitted lane lines.

In this paper, we randomly select three images from each scenario and analyze the fitting effects of the detected lane lines to show the superiority of the proposed lane line fitting approach. We fit four lanes within the field of view of the current driving lane with 30-pixel lines by preprocessing the input image with cropping and normalization operations and loading the trained model; the obtained lane line fitting results are shown in Fig 13. The Nb_SINet model fits lines relatively well in the normal, crowded, shadow, night, arrow, and dazzle light scenarios, while the fitting effect still needs to be improved when there are no lines, curves, or crossroads.

## Conclusions

In this paper, we introduced a real-time lane detection model called Nb_SINet that fuses Non-bt-1D-SRC and an IAP to address the problem regarding the poor lane line detection accuracy and real-time performance of ERF-PSPNet in multiscenario environments. We adopted a Non-bt-1D-SRC in the encoder, which incorporates multiple features after performing asymmetric convolution to enhance the *mIOU* achieved during SS. An improved feature pyramid network was used for the decoding phase, which introduces an attention mechanism and uses the AP module to obtain rich contextual information. The contributions of this paper are as follows: (1) we propose a Non-bt-1D-SRC module to solve the problem regarding the lack of correlated feature information between adjacent convolutional layers. (2) The IAP module extracts rich contextual information. In a comparison with ENet_LGAD, SIM_CycleGAN+ERFNet, ERFNet-E2E, ERFNet_VP and ERFNet-HESA, the experimental results show that our model improved the *F1* values in five scenarios: normal, shadow, arrow, dazzle light, and no line. The mean *F1* is also higher for the nine tested scenarios. Meanwhile, our model has fewer parameters and the shortest runtime.

In the future, we need to further enhance the lane line fitting effect yielded in the curve scenario. Next, we will consider using the biarc spline function for fitting purposes. In the crossroad scenario, the number of *FPs* is largest, mainly because the crossroad data are not finely labeled, and the number of lanes is the greatest. In this paper, we only considered the four left and right lane lines from where the vehicle was located. A further step will be to focus on multiple lanes at a crossroad and carefully annotate the data.

## Supporting information

S1 Fig Auxiliary training loss. The cross-entropy loss of the auxiliary trainer is used as the auxiliary loss, which solves the problem of gradient disappearance.
(TIF)

S2 Fig Lane forecast loss. Lane prediction loss is used to evaluate the quality of lane prediction.
(TIF)

S3 Fig SS loss. Semantic segmentation is used to segment the image background and four lane lines, and the calculation of cross-entropy loss can evaluate the effect of SS.
(TIF)
