# Supplementary figures and images for: Real-time lane detection model based on non bottleneck skip residual connections and attention pyramids
Source: PLoS One. 2021 Oct 19;16(10):e0252755. doi: 10.1371/journal.pone.0252755 (PMC8525742; doi:10.1371/journal.pone.0252755)

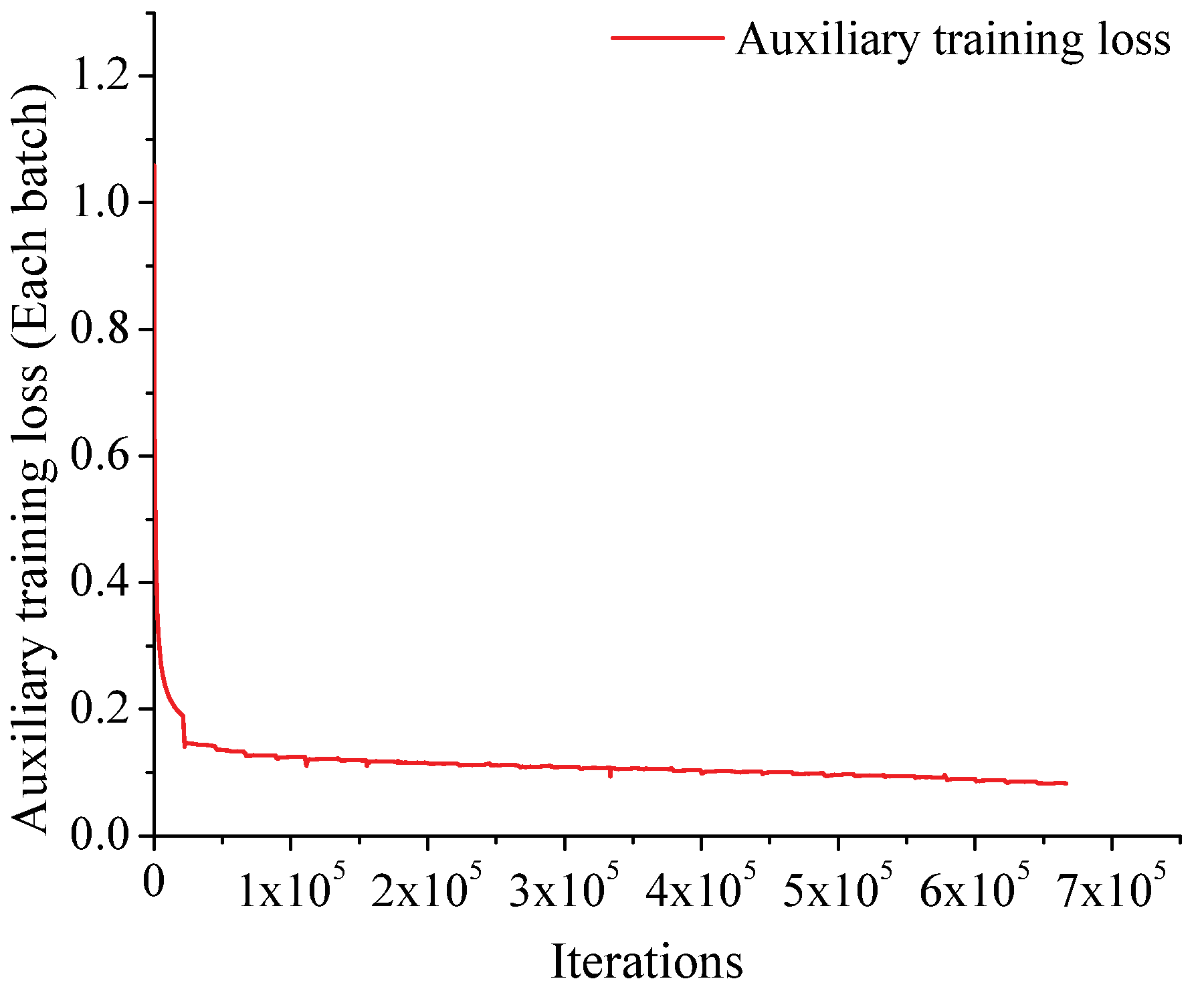

Supplement: S1 Fig — The cross-entropy loss of the auxiliary trainer is used as the auxiliary loss, which solves the problem of gradient disappearance. (TIF) [file pone.0252755.s001.tif]

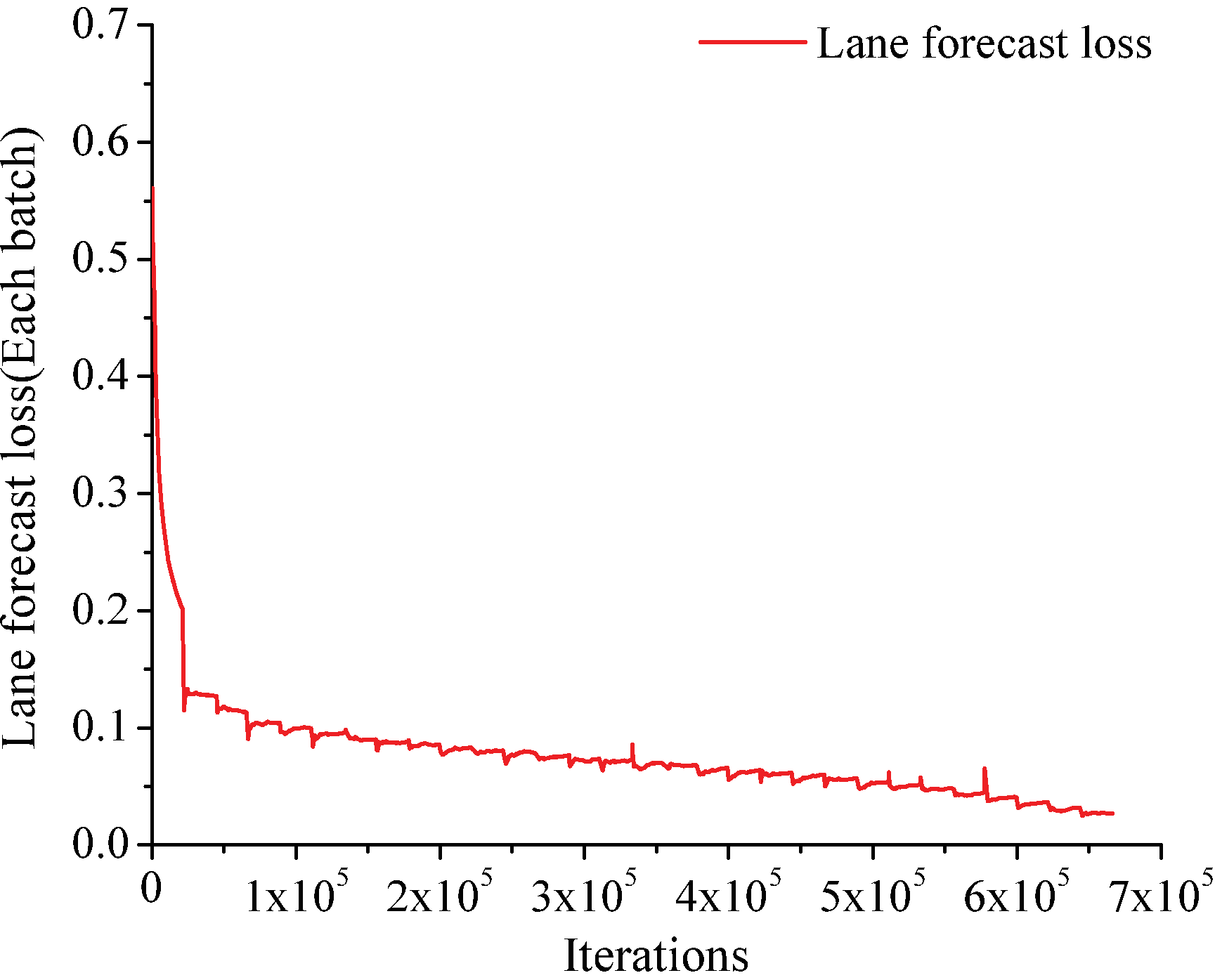

Supplement: S2 Fig — Lane prediction loss is used to evaluate the quality of lane prediction. (TIF) [file pone.0252755.s002.tif]

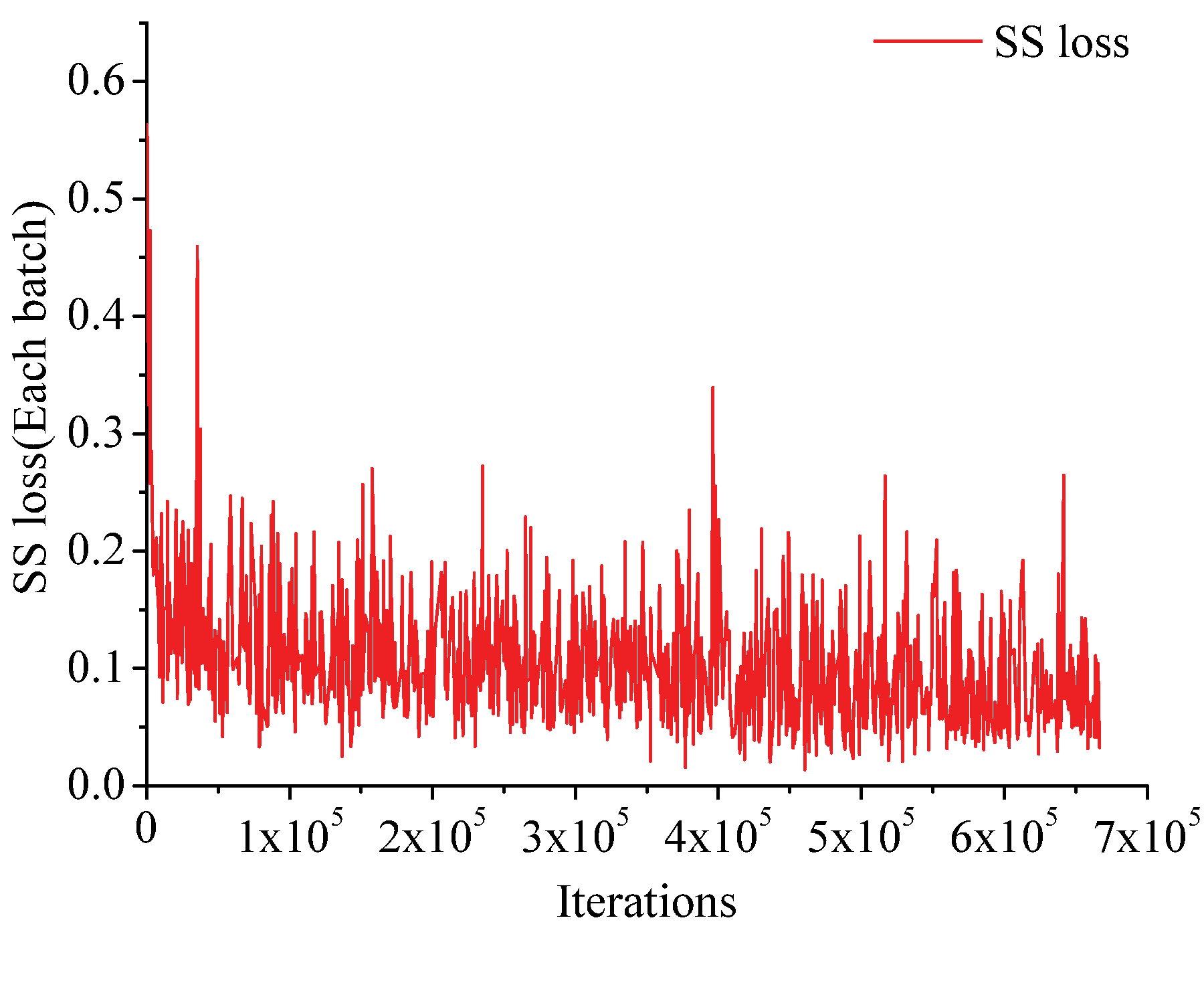

Supplement: S3 Fig — Semantic segmentation is used to segment the image background and four lane lines, and the calculation of cross-entropy loss can evaluate the effect of SS. (TIF) [file pone.0252755.s003.tif]
